# Synaptic integration and NMDA spikes in a layer 5 pyramidal neuron model

**DOI:** 10.1186/1471-2202-12-S1-P301

**Published:** 2011-07-18

**Authors:** Matteo Farinella, Padraig Gleeson, Daniel CT Ruidt, Angus R Silver

**Affiliations:** 1 Department of Neuroscience, Physiology and Pharmacology, University College London, London WC1E 6BT, UK

## 

Several central neurons exhibit regenerative dendritic events, which are believed to increase the computational power of the cell [1]. NMDA receptor-mediated spikes [2] have been triggered *in-vitro* in thin dendrites [3] of all the main classes of cortical pyramidal neurons. They are characterized by a local depolarization of approximately 40-50 mV and a duration that ranges from 50 to 100 ms, depending on the input strength [4] and the other currents recruited. This long duration suggests that the temporal integration window is significantly longer than the membrane time constant which may be important during the low firing frequencies observed in some cortical regions such as barrel cortex. Moreover, their dependence on glutamate binding suggests that they are spatially restricted to the synaptic input zone, unlike dendritic spikes mediated by Na^+^ channels. These features make them particularly well suited for amplifying clustered synaptic input [5], which is one of the essential requirements for the pyramidal neuron to act as a two layer network [6]. They have also been suggested to play a key role in burst detection [7] and plasticity [8]. However, because of their distal location within fine dendritic structures, it is extremely difficult to investigate their properties experimentally. To circumvent this we have investigated the properties of synaptic integration and NMDA spike generation using a biologically detailed multi-compartmental modelling approach. We have used the L5 pyramidal cell model developed by Larkum^3^ and colleagues as our basic model. This was translated into NeuroML [9], making more accessible for use with modelling tools such as neuroConstruct [10]. NMDA spikes were activated with coincident synaptic input onto specific apical branches and defined with a threshold criterion. We determined the input-output relationship of different dendritic branches by quantifying the relationship between the probability of generating an NMDA spike and number of synapses activated on the branch. Consistent with previous experimental studies we found that 10-20 synapses were necessary to trigger an NMDA spike. On average, coincident NMDA spikes in 13 different apical dendritic branches were necessary to fire a somatic action potential with a 0.5 probability. However, the number of NMDA spikes required to trigger a somatic action potential was significantly reduced in the presence of background excitatory synaptic input (1500 inputs at 0.85Hz). These results suggest that NMDA spikes could have a greater contribution to integrating synaptic input in active networks than observed in acute slices where there is little ongoing synaptic activity.
